# The multifaceted impact of anxiety and depression on patients with rheumatoid arthritis

**DOI:** 10.1186/s41927-019-0092-5

**Published:** 2019-10-28

**Authors:** Steve Peterson, James Piercy, Stuart Blackburn, Emma Sullivan, Chetan S. Karyekar, Nan Li

**Affiliations:** 1 Janssen Global Services, Horsham, PA USA; 2 Adelphi Real World, Macclesfield, UK; 3 Manchester Metropolitan University, Manchester, UK

**Keywords:** Rheumatoid arthritis, Depression, Anxiety, Comorbidity

## Abstract

**Background:**

The prevalence of mood disturbances such as anxiety and depression is greater in rheumatoid arthritis (RA) patients than in the general population. Given this association, the primary aim of this study was to assess the incremental impact of anxiety or depression on patients with RA from the United States of America (USA) and Europe, independent of the impact of the underlying RA disease.

**Methods:**

Rheumatologists (*n* = 408) from the USA and 5 European countries completed patient record forms for a predetermined number of RA patients who consulted consecutively during the study period; these patients completed patient-reported questionnaires. Descriptive statistics and multivariate regression were used to investigate the relationship between anxiety and depression with treatment and economic outcomes in RA patients.

**Results:**

Of 1015 physician and patient pairs who completed all relevant questionnaire sections, 390 (38.4%) patients self-reported anxiety or depression, while 180 (17.7%) patients were reported to have anxiety or depression by their physicians. Controlling for age, gender, body mass index and clinical factors (flaring and severity), multiple regression analyses suggested that patients with anxiety or depression more often experienced treatment dissatisfaction (odds ratio [OR] 2.28; *P* < .001), had greater impairment in work (coefficient [β] = 11.82; *P* = .001) and usual activity (β = 14.73; *P* < .001), greater disability (β = .35; *P* < .001), and more often reported unemployment (OR 1.74; *P* = .001). Multinomial logistic regression revealed discordance between physician and patient satisfaction with treatment. For patients reporting anxiety or depression, physicians were more often satisfied with achievement of current disease control than patients (relative risk ratio 2.19; *P* = .002).

**Conclusion:**

Concomitant anxiety or depression was associated with a significant incremental impact on the health-related quality of life and economic aspects of life of patients with RA. In light of observed differences between physician recognition of patient anxiety and/or depression versus patient reporting of anxiety and/or depression symptoms, further research is warranted to develop optimal screening and management of depression and anxiety in patients with RA.

## Background

Rheumatoid arthritis (RA) is a chronic autoimmune inflammatory disease that has significant and diverse impact on patients’ health-related quality of life (HRQoL) [1]. Patients with RA experience fatigue, chronic pain, restriction of activities and increased rates of disability. Not surprisingly, patients with RA have an increased prevalence of mood disturbances such as anxiety or depression [2].

A systematic review and meta-analysis of 72 studies performed in various countries revealed an estimated prevalence of depression of 16.8% [3]; the prevalence from individual studies ranged from 9.5% [4] to 41.5% [5]. In comparison, the 12-month prevalence of depression in the general adult population in the United States of America (USA) is 6.6% [6], which is half or less than the estimated prevalence observed in RA. Comorbid depression among RA patients is associated with a burden of illness that is incremental to the burden of illness of RA alone, and is associated with poor clinical and treatment outcomes. For example, RA patients with comorbid depression experience increased pain levels [7, 8] regardless of disease activity [9]. Comorbid depression is an independent risk factor for cardiovascular disease [10], myocardial infarction [11] and all-cause mortality [12]. Depression may also adversely affect treatment outcomes [13–15]. In addition, depression is associated with increased healthcare costs [16], work disability [17] and unemployment [18]. Despite the high prevalence and association with poor outcomes, optimal management of comorbid depression in RA is not fully characterized in the medical literature, is not prioritized in major treatment guidelines, and therefore may not be appropriately recognized and managed by all healthcare providers [19–21].

When compared with depression, comorbid anxiety appears to have received even less attention in the RA literature [22, 23]. Some studies have indicated that anxiety is common in patients with RA. A Canadian study reported that the incidence of depression was 46% higher in patients with RA compared to a matched control group, while the incidence of anxiety was 24% higher in patients with RA compared to a matched control group [24]. A separate study conducted in Hong Kong reported a 16% lifetime prevalence of anxiety disorders in patients with RA [4]. Similar to depression, comorbid anxiety contributes an additional burden to patients with RA; in this population, anxiety is also associated with lower HRQoL [18] and suboptimal treatment responses [15].

Given the associations between anxiety or depression and RA, and the interactions between mental health and disease activity, the primary objective of this study was to assess the incremental impact of anxiety or depression on the HRQoL and economic aspects of life among patients with RA from the USA and Europe, while controlling for the underlying RA disease severity or activity. A secondary objective was to assess the physician- and patient-reported prevalence of anxiety or depression, and to identify any potential discordance between physicians and patients regarding the reporting of these conditions.

## Methods

### Data source

Data for this study were drawn from the Adelphi Real World RA Disease Specific Programme (DSP). DSPs are large, multinational matched point-in-time physician and patient surveys that are conducted through a broad sample of real clinical practice settings. DSPs collect patient and physician data on: Patient characteristics, current and past treatments, current disease activity and control, and a broad array of patient satisfaction and patient patient-reported measures. For this study, data were drawn from the Adelphi RA DSP conducted between January and June 2014 in the USA and 5 European countries (France, Germany, Italy, Spain and the United Kingdom). A complete description of the survey methods has been previously published and validated [25–27].

In each country, physicians completed a record form for 8 consecutive patients with a physician-confirmed diagnosis of RA who visited them for routine non-emergency care for their RA during the data collection period. This physician-reported form contained detailed questions on patient demographics; current and historical clinical status including physician’s assessment of current severity and whether they thought the patient was improving, stable or deteriorating; whether in their opinion the patient was currently experiencing a flare and also how many flares there had been in the previous 12 months; current and prior treatment history; and whether the patient was currently experiencing concomitant conditions (including anxiety and depression).

Each patient for whom the physician completed a form was then invited to complete a patient-reported questionnaire. Of the 1035 consulting patients, 1015 patients filled out the complete questionnaire and therefore 20 patients had to be excluded from the analysis owing to missing data. Each patient who agreed to participate was asked to provide appropriate informed consent. Patient-reported questionnaires contained detailed questions on demographics, satisfaction with current treatment, attitudes toward their condition and the impact of their disease including validated Patient Reported Outcome surveys. Patients also reported current levels of pain on a scale of 1 to 10, where 1 represented “no pain” and 10 represented “the worst pain imaginable”.

Data collection was performed in accordance with the European Pharmaceutical Marketing Research Association guidelines [28]. As such, ethics committee approval was not required. Each survey was performed in full accordance with relevant legislation at the time of data collection including the US Health Insurance Portability and Accountability Act 1996 [29], and Health Information Technology for Economic and Clinical Health Act legislation [30]. Physicians were compensated according to fair rates for market research. Patients consented to provide anonymized and aggregated information for analysis and publication but were not paid. Patients were included in the analysis if both the reporting physician and patient completed their respective questions relating to anxiety and depression.

### Participating physicians and patients

Physicians were recruited from publicly available lists in each country. Physicians were eligible to participate in this survey if they were personally responsible for assessment, treatment decisions and management of > 7 RA, > 1 ankylosing spondylitis, > 1 non-radiographic axial spondyloarthritis and > 2 psoriatic arthritis patients in a typical month. Physicians who had qualified less than 2 years prior to the commencement of the survey were also excluded. Qualifying physicians were then instructed to select their 8-patient samples as: Consecutive patients starting at a pre-defined date meeting the following inclusion criteria: Adults ≥18 years of age, physician-confirmed diagnosis of RA and not currently involved in a clinical trial.

### Measures of anxiety and depression

The DSP captured both physician-reported comorbid diagnosis of, and patient-reported current symptoms of anxiety and depression. In the physician-completed patient report form, physicians were asked to report any concomitant conditions that the patient had, specifically asking “Does the patient currently suffer from any concomitant conditions?”; anxiety and depression were listed separately in a prespecified list of conditions. For analysis, these responses were combined into a single variable capturing any mention of physician-reported anxiety, depression or both (hereafter shown as “anxiety/depression”) for comparability with the patient reports. Patient-reported anxiety or depression was captured through the EuroQol (EQ-5D-3L) questionnaire [31], specifically the “anxiety/depression” domain. In this domain, patients responded by choosing 1 of 3 options (“I am not anxious or depressed”/“I am moderately anxious or depressed”/“I am extremely anxious or depressed”). For the analyses presented here, no distinction was made between patients who responded as being “moderately” or “extremely” anxious or depressed; these patients were combined into a single group. Patients were thus categorized as being either “negative” or “positive” for these conditions. Patient-reported anxiety or depression (hereafter also shown as “anxiety/depression”) was used as the primary indicator of anxiety/depression for analysis.

### Outcomes

The correlation between anxiety or depression and Health Assessment Questionnaire Disability Index (HAQ-DI) score, productivity, daily activity impairment, employment status, and patient-reported and physician-reported satisfaction was examined.

The HAQ-DI is composed of 20 items in 8 categories (dressing and grooming, arising, eating, walking, hygiene, reach, grip and activities) [32, 33]. Work productivity was assessed using the Work Productivity and Activity Impairment (WPAI) questionnaire, which assesses absenteeism (work time missed), presenteeism (impairment while at work) and overall impairment in work productivity (combination of absenteeism and presenteeism). The overall impairment was calculated on a scale of 0–100, with a higher score indicting a greater degree of impairment. Patients were also asked to indicate the impairment in daily activities attributed to their health problems over the previous 7 days [32].

Regarding satisfaction with current treatment; physicians were asked to assess their satisfaction with current control of the patients’ RA, while patients were asked about their satisfaction with current treatment. Response options for both physicians and patients were identical: “satisfied”, “not satisfied but I believe this is the best control that can be realistically achieved”, and “not satisfied, and I believe better control can be achieved”.

### Statistical analysis

Descriptive statistics are presented throughout, including reported rates of anxiety/depression from both the physicians and patients. Bivariate analysis was performed to assess any unadjusted differences in patient demographics, clinical status and clinical measurements for patients reporting anxiety/depression. Mann-Whitney’s rank sum tests were used when the outcome variable was continuous or ordinal, Pearson’s chi-squared tests for dichotomous outcome variables and Fisher’s exact tests for categorical variables with more than 2 categories.

Multivariate linear analysis was used to assess the association of patient-reported anxiety/depression with the following outcomes: overall work impairment, daily activity impairment and HAQ-DI score. Multivariate logistic regression analysis was used for employment status and patient dissatisfaction with current treatment approach. Multivariate multinomial logistic regression was used to analyze patient and physician concordance on satisfaction with current treatment approach, grouping responses into 3 distinct categories (“both agree”/“patient satisfied but physician dissatisfied”/“physician satisfied but patient dissatisfied”). Patient and physician agreement was used as the reference level in the model. Model coefficients are reported for linear models, odds ratios (ORs) for logistic models and relative risk ratios (RRRs) for multinomial logistic models. Confounding factors controlled for in the models were the following: patient age, region (the USA or Europe), gender, body mass index (BMI), current severity as stated by the physician, number of flares in the last 12 months and physician global assessment. For all models, standard errors were adjusted using the clustered sandwich estimator to allow for intragroup correlation for each reporting physician, relaxing the usual requirement that the observations be independent (i.e. the patients are independent across physicians but not necessarily for each physician).

The significance level for all analyses was set at 5%, with all tests being 2-sided. All data were analyzed using Stata software, release 15 [34].

## Results

A total of 408 rheumatologists participated in the RA DSP. Physician-completed record forms with corresponding patient-reported questionnaires were available from a total of 1035 patients with RA. Of these patients, 1015 completed the anxiety/depression domain of the EQ-5D-3L (*n* = 408, USA; *n* = 607, Europe); these patients were the focus of our analyses.

### Prevalence of anxiety or depression

Using questionnaires completed by both physicians and patients, we were able to compare physician-reported and patient-reported anxiety/depression, and estimate the degree of discrepancy between physicians and patients. Of 1015 physician and patient pairs, 390 (38.4%) patients reported that they had anxiety/depression, while 180 (17.7%) patients were reported to have anxiety/depression by their physicians (*P* < .001, paired McNemar’s test). In 681 (67.1%) cases, both the physician and patient agreed with the assessment of anxiety/depression, while in patients with patient-reported anxiety/depression (*n* = 390), 272 (69.7%) physicians did not report anxiety/depression. In contrast, of patients not reporting anxiety/depression (*n* = 625), physicians reported anxiety/depression in 62 (9.9%) cases. This discordance between physicians and patients was observed in both the USA and Europe (Table 1).

**Table 1:** Prevalence of anxiety/depression, and patient and physician concordance

		Physician-reported, n (%)			*P*-value
Overall		Total	Anxiety/depression	No anxiety/depression	<.001^a^
Patient-reported	Total	1015 (100)	180 (17.7)	835 (82.3)	
	Anxiety/depression	390 (38.4)	118 (11.6)	272 (26.8)	
	No anxiety/depression	625 (61.6)	62 (6.1)	563 (55.5)	
Europe		Total	Anxiety/depression	No anxiety/depression	<.001^a^
Patient-reported	Total	607 (100)	96 (15.8)	511 (84.2)	
	Anxiety/depression	248 (40.9)	67 (11.0)	181 (29.8)	
	No anxiety/depression	359 (59.1)	29 (4.8)	330 (54.4)	
USA		Total	Anxiety/depression	No anxiety/depression	<.001^a^
Patient-reported	Total	408 (100)	84 (20.6)	324 (79.4)	
	Anxiety/depression	142 (34.8)	51 (12.5)	91 (22.3)	
	No anxiety/depression	266 (65.2)	33 (8.1)	233 (57.1)	

Europe, France, Germany, Italy, Spain and the United Kingdom; USA, the United States of America

^a^Paired McNemar’s test comparing patient-reported anxiety/depression to physician-reported anxiety/depression

### Univariate analyses

#### Demographics

We compared the demographics of patients reporting anxiety/depression with those who did not report these conditions. We observed that patients who reported anxiety/depression were older (mean age 56.7 vs 54.3 years; *P* = .012), female (77.4% vs 69.0%; *P* = .004) and had more severe RA (moderate, 40.0% vs 22.1%; severe, 7.9% vs 1.3%; *P* < .001) with longer disease duration (mean 8.7 vs 6.7 years; *P* < .001). These patients also experienced more severe pain (mean score 4.1 vs 2.8; *P* < .001) on a scale of 1 to 10. A greater proportion of these patients had received biologic therapy (52.6% vs 42.2%; *P* = .002); with 90% receiving continuing therapy in both groups (Table 2). There were no differences in either discontinuation rates or patients on break from therapy (*p* = 0.636).

**Table 2:** Patient demographics

	Overall, *N* = 1015	No self-reported anxiety/depression, *n* = 625	Self-reported anxiety/depression, *n* = 390	*P-*value (^a^test)
Patient age, years				
Mean (SD)	55.2 (14.1)	54.3 (14.6)	56.7 (13.1)	.012 (MW)
Gender, n (%)				
Female	733 (72.2)	431 (69.0)	302 (77.4)	.004 (FE)
Years since RA diagnosis				
Mean, years (SD)	7.5 (7.4)	6.7 (7.0)	8.7 (7.9)	.001 (MW)
^b^Missing, n	69	38	31	
Current severity level, n (%)				
Mild	682 (67.2)	479 (76.6)	203 (52.1)	
Moderate	294 (29.0)	138 (22.1)	156 (40.0)	
Severe	39 (3.8)	8 (1.3)	31 (7.9)	<.001 (MW)
Current pain level (1–10)				
Mean (SD)	3.3 (2.0)	2.8 (1.7)	4.1 (2.2)	<.001 (MW)
On biologic therapy, n (%)				
Receiving biologic therapy/on break but expected to restart	469 (46.2) 20 (2.0)	264 (42.2) 10 (1.6)	205 (52.6) 10 (2.6)	.002 (FE)
Discontinued biologic, n (%)	30 (3.0)	16 (2.6)	14 (3.6)	0.636 (PC)

*FE* Fisher’s exact test; *MW* Mann-Whitney’s U test; *PC* Pearson’s Chi-square test; *RA* rheumatoid arthritis; *SD* standard deviation

^a^Indicates statistical test performed

^b^Number of patients for whom data were not reported

#### Association between anxiety or depression and patient outcomes

A lower proportion of patients who reported anxiety/depression were in remission (40.0% vs 57.0%; *P* < .001), or stable or improving (71.8% vs 90.2%; *P* < .001). In addition, a greater proportion of these patients were experiencing flares (19.2% vs 9.3%; *P* < .001). Patients who reported anxiety/depression also had higher Disease Activity Score-28 joint count (DAS28) scores (mean score 4.0 vs 3.4; *P* < .001) and higher disability (mean HAQ-DI score, 1.1 vs 0.6; *P* < .001) (Table 3).

**Table 3:** Impact of anxiety/depression in patients with RA

	Overall, *N* = 1015	No self-reported anxiety/depression, *n* = 625	Self-reported anxiety/depression, *n* = 390	*P-*value (^a^test)
Clinical measures				
Current flare status, n (%)				
No	860 (86.9)	553 (90.7)	307 (80.8)	
Yes	130 (13.1)	57 (9.3)	73 (19.2)	<.001 (FE)
^b^Missing, n	25	15	10	
DAS28-ESR				
Mean score (SD)	3.6 (1.4)	3.4 (1.3)	4.0 (1.5)	<.001 (MW)
^b^Missing, n	348	215	133	
Current disease progression, n (%)				
Unstable/deteriorating	167 (16.9)	60 (9.8)	107 (28.2)	
Stable	523 (52.8)	339 (55.6)	184 (48.4)	
Improving	300 (30.3)	211 (34.6)	89 (23.4)	<.001 (PC)
^b^Missing, n	25	15	10	
Is patient currently in remission?, n (%)				
No	503 (49.6)	269 (43.0)	234 (60.0)	
Yes	512 (50.4)	356 (57.0)	156 (40.0)	<.001 (FE)
Employment and impairment				
Patient’s employment status, n (%)				
Unemployed	553 (55.8)	301 (49.1)	252 (66.7)	
Employed	438 (44.2)	312 (50.9)	126 (33.3)	<.001 (FE)
^b^Missing, n	24	12	12	
Percent overall work impairment due to problem (range 0–100)				
Mean (SD)	23.3 (24.4)	18.3 (21.7)	37.5 (26.3)	<.001 (MW)
^b^Missing, n	684	380	304	
Percent activity impairment due to problem (range 0–100)				
Mean (SD)	33.4 (25.8)	25.0 (22.4)	47.1 (25.1)	<.001 (MW)
^b^Missing, n	78	44	34	
^c^Retired/unemployed due to condition, n (%)				
Not due to RA	92 (68.7)	46 (80.7)	46 (59.7)	
Due to RA	42 (31.3)	11 (19.3)	31 (40.3)	.014 (FE)
^b^Missing, n	881	568	313	
Ever changed job due to condition?, n (%)				
No	804 (90.1)	527 (93.4)	277 (84.5)	
Yes	88 (9.9)	37 (6.6)	51 (15.5)	<.001 (FE)
^b^Missing, n	123	61	62	
Disability				
HAQ-DI				
mean HAQ-DI score (SD)	0.8 (0.7)	0.6 (0.6)	1.1 (0.7)	<.001 (MW)
^b^Missing, n	44	24	20	

*DAS28-ESR* disease activity score-28 joint count–erythrocyte sedimentation rate; *FE* Fisher’s exact test; *HAQ-DI* Health Assessment Questionnaire Disability Index; *MW* Mann-Whitney’s U test; *PC* Pearso n’s chi-squared; *RA* rheumatoid arthritis; SD, standard deviation

^a^Indicates statistical test performed

^b^Number of patients for whom data were not reported

^c^For patients who retired below the retirement age of 65 years. Data available only from patients who initially responded as being retired or unemployed

A lower proportion of patients who reported anxiety/depression were employed (33.3% vs 50.9%; *P* < .001). A greater proportion of these patients experienced impairment if working (WPAI overall work impairment mean score 37.5% vs 18.3%; *P* < .001), were retired (40.3% vs 19.3%; *P* = .014) or changed jobs (15.5% vs 6.6%; *P* < .001) due to their condition. Overall daily activity impairment was also greater in these patients (mean score 47.1% vs 25.0%; *P* < .001) (Table 3).

A lower proportion of patients who reported anxiety/depression were satisfied with their current RA treatment (61.8% vs 83.4%; *P* < .001). Concurrently, a lower proportion of physicians were satisfied with disease control in patients who reported anxiety/depression (58.7% vs 79.0%; *P* < .001). We observed lower concordance on treatment satisfaction between physicians and patients for patients who reported anxiety/depression (75.4% vs 82.9%; *P* = .009) (Table 4).

**Table 4:** Treatment satisfaction, and patient and physician concordance

	Overall, *N* = 1015	No self-reported anxiety/depression, *n* = 625	Self-reported anxiety/depression, *n* = 390	*P-*value (^a^test)
Physician-reported satisfaction with current control of patient condition, n (%)				
Not satisfied	292 (28.8)	131 (21.0)	161 (41.3)	
Satisfied	723 (71.2)	494 (79.0)	229 (58.7)	<.001 (FE)
Patient-reported satisfaction with current treatment of condition, n (%)				
Not satisfied	231 (24.8)	96 (16.6)	135 (38.2)	
Satisfied	701 (75.2)	483 (83.4)	218 (61.8)	<.001 (FE)
^b^Missing, n	83	46	37	
Patient-physician concordance on satisfaction with current treatment, n (%)				
Both agree	746 (80.0)	480 (82.9)	266 (75.4)	
Physician satisfied (not patient)	77 (8.3)	37 (6.4)	40 (11.3)	
Patient satisfied (not physician)	109 (11.7)	62 (10.7)	47 (13.3)	.009 (PC)
^b^Missing, n	83	46	37	

*FE* Fisher’s exact test; *PC* Pearson’s chi-squared

^a^Indicates statistical test performed

^b^Number of patients for whom data were not reported

### Multivariate analyses

We performed multiple regression analysis to assess the association of anxiety/depression with various outcomes while controlling for several potential confounders including patient age, region (the USA or Europe), gender, BMI, current disease severity as stated by the physician, number of flares in the last 12 months and physician global assessment. Among patients who reported anxiety/depression, we observed higher impairment of both work (coefficient [β] = 11.82, 95% confidence interval [CI] 5.02–18.62; *P* = .001) and usual activity (β = 14.73, 95% CI 11.34–18.11; *P* < .001), greater disability using the HAQ-DI (β = 0.35, 95% CI 0.25–0.45; *P* < .001), greater likelihood of unemployment (OR 1.74; 95% CI 1.25–2.42; *P* = .001) and greater dissatisfaction with treatment (OR 2.28, 95% CI 1.54–3.37; *P* < .001) (Table 5). Multinomial logistic regression showed that for patients reporting anxiety/depression relative to those without, the relative risk for the physician being satisfied and the patient not versus both patient and physician agreeing (reference level) increased 2.2 times (RRR 2.19, 95% CI 1.32–3.63; *P* = .002). However, in the same model, there was no significant association with patient (but not physician) satisfaction versus the reference level of both patient and physician agreeing for patients reporting anxiety/depression.

**Table 5:** Multiple regression analysis

	^a^n	Result	*P-*value	95% CI
^b^Model coefficients				
HAQ-DI	947	0.349	<.001	0.247–0.451
Percent overall work impairment	323	11.819	.001	5.019–18.618
Percent activity impairment	912	14.728	<.001	11.341–18.115
^c^Odds ratios				
Unemployment	966	1.737	.001	1.248–2.417
Patient-reported dissatisfaction	913	2.278	<.001	1.540–3.368
^d^Relative risk ratio				
Both patient and physician agree	[Reference category in multinomial logistic regression]			
Physician satisfied only	913	2.193	.002	1.325–3.629
Patient satisfied only	913	0.831	.443	0.517–1.334

All models controlled for age, region (the USA or Europe), patient sex, body mass index, current severity as stated by the physician, number of flares in the last 12 months and physician global assessment

*CI* confidence interval; *HAQ-DI* Health Assessment Questionnaire Disability Index; *T2T* treat-to-target

^a^Sample size for regression model

^b^The coefficient indicates the unit increase of parameter of interest for patients reporting anxiety/depression on the EQ-5D-3L domain compared with those not reporting any anxiety/depression

^c^The odds ratio indicates the odds of the parameter of interest occurring for patients reporting anxiety/depression on the EQ-5D-3L domain compared with those not reporting any anxiety/depression

^d^The relative risk ratio indicates the factor increase in the relative risk of only the physician being satisfied with current treatment (not the patient) for patients reporting anxiety or depression on the EQ-5D-3L domain compared with those not reporting any anxiety or depression

## Discussion

Comorbid depression and anxiety present additional burden of illness above and beyond the burden from core RA symptoms alone. Consistent with previous literature [3], this analysis of data collected in the Adelphi RA DSP revealed a high prevalence of anxiety or depression in patients with RA compared to the prevalence reported in the general population. We also observed that patient-reported anxiety/depression was associated with poorer outcomes across a variety of measures including patient function, likelihood of remission, employment, work and daily activity impairment, and treatment satisfaction.

Prevalence of anxiety/depression (combined) based on patient self-reported assessments was 38.4% in this patient cohort. This rate is consistent, albeit at the higher end of the ranges between 9.5% [4] and 41.5% [5] reported in previous studies, which included the diagnosis of depression using an established instrument for depression diagnosis, but did not include the diagnosis of anxiety. In our study, anxiety or depression were self-reported on the basis of the patient’s own assessment by the EQ-5L-3D. In addition, our study indicated that the physician-reported prevalence of anxiety/depression was 17.7%, a figure that is consistent with a previous meta-analysis estimate [3], suggesting a substantial unrecognition by physicians in patients who self-report symptoms of anxiety or depression. In a study of patients with moderate-to-severe RA, only 19% of the patients discussed depression with their rheumatologist, and in all these cases, the discussion was initiated by the patients and was not queried by the physician [35]. It is therefore likely that a number of patients may not volunteer symptoms or diagnosis of anxiety or depression with their physicians who treat RA. Enabling patients to describe important symptoms of comorbidities such as anxiety and depression or eliciting previous diagnosis or screening for these comorbidities would be important to manage patients to attain better clinical and patient outcomes.

The EQ-5D-3L instrument is not designed as a measure of disease prevalence of depression or anxiety. It requires the patient to self-report whether they are currently anxious or depressed. Also, no distinction is made between anxiety and depression in the tool. This is in contrast to the physician-reported questions, where anxiety and depression were treated individually and the physician could choose one or both conditions. For the analyses presented here, physicians reporting anxiety, depression, or both anxiety and depression, were classified as reporting anxiety/depression, thus improving comparability with patient-reported anxiety/depression. Although anxiety and depression are distinct conditions, there is considerable diagnostic overlap between generalized anxiety disorder and major depressive disorder [36]. The difference in instruments used in this survey to elicit information on anxiety and depression from physicians compared to patients may in part explain differences in prevalence of patient-reported depression/anxiety and physician–ascribed comorbid depression or anxiety. While differences in instruments used to elicit physician-ascribed and patient-reported anxiety and depression may explain part of the disparity in our physician- and patient-reported prevalence, earlier research has documented under-reporting of anxiety and depression on the part of patients [35]. Considering this, and given the evidence regarding impact of comorbid depression/anxiety on patient-reported outcomes, enhancements in clinical screening and management of comorbid anxiety/depression may be beneficial in the management of RA.

The interactions between mental comorbidities and RA disease activity are multifaceted and likely mutually influential, or bidirectional [37]. For example, a study in Japanese patients with RA revealed a significant association between both depression score and C-reactive protein levels with pain [38]. The increased levels of circulating proinflammatory cytokines in patients with RA [39] lead to alterations in immune function, which in turn affected psychological states [40]. Given this bidirectionality, we controlled for several factors in the multiple regression analyses including age, gender, BMI and clinical factors such as flaring and severity to understand the association. Our research indicates that the presence of anxiety and/or depression in RA patients resulted in more patients experiencing treatment dissatisfaction, having greater work impairment with higher unemployment and having greater disability. While a causal relationship cannot be inferred from these results, we nevertheless observed a significant association between anxiety or depression and various outcomes described above. These outcomes are important from a clinical perspective, as well as management of the population of RA patients by a payer organization. It is possible that other socio-demographic and treatment factors such as income, education, smoking status, alcohol consumption, and presence of or treatment for mood disorders could also impact these outcomes. As these variables were not collected in this study, further research that could incorporate and account for a wider range of covariates is necessary to establish the causal role of anxiety and depression on outcomes in RA patients.

As this was an analysis of real-world data, there are a number of strengths and limitations. We collected real-world data at the time of consultation by the physician as part of a routine clinic visit and no additional investigations, visits or tests were performed as part of the survey for any reason. The fieldwork materials permitted collection of extensive information from both physicians and patients at the same time, eliminating recall bias. This allowed assessment of the discrepancy between patient-reported and physician-reported anxiety/depression. While minimal inclusion criteria governed the selection of participating physicians, the physician sample may have been influenced by willingness to complete the survey. The patient sample may have been influenced by study design whereby physicians were instructed to select consecutive patients from a given date. This sampling rule may have biased towards more frequently visiting patients, who may have been more severely affected than the general population. Furthermore, it is also possible that patients who are anxious or depressed may be more or less likely to participate in research. We noted that for patients filling out a self-completion form in this study, the physician-reported prevalence of anxiety/depression was 17.9% compared to a prevalence of 14.3% in patients who did not fill out a form (*P* < 0.01). However, because the core analyses presented characterize the association between patient-reported anxiety/depression and various outcomes, this discrepancy does not have any bearing on the internal validity of the analyses conducted. As such, this difference should not detract from the core conclusion of this analysis that anxiety or depression warrant treatment attention in their own right for patients being treated with RA.

## Conclusions

In summary, this analysis of real-world data highlighted the impact of anxiety or depression on patient-reported and economic outcomes in RA. Longitudinal and prospective studies are necessary to assess the impact of identification and optimization of treatment of mental disorders associated with RA. In the meantime, rheumatology healthcare providers and their patients may benefit from collaboration with mental and primary healthcare providers to optimally manage this comorbidity.

## Data Availability

The data that support the findings of this study are available from Adelphi Real World but restrictions apply to the availability of these data, which were used under license for the current study, and so are not publicly available. Data are, however, available from the authors upon reasonable request and with permission of Adelphi Real World.

## Abbreviations

BMI: Body mass index
CI: Confidence interval
DAS28: Disease activity score-28 joint count
DSP: Disease specific programme
EQ-5D-3L: EuroQol EQ-5D-3L Descriptive System
HAQ-DI: Health Assessment Questionnaire Disability Index
HRQoL: Health-related quality of life
OR: Odds ratio
RA: Rheumatoid arthritis
RRR: Relative risk ratio
T2T: Treat-to-target
USA: the United States of America
WPAI: Work productivity and activity impairment
β: Coefficient
